# The Relationship between Neutralization Techniques and Induced Abortion

**Published:** 2014-04

**Authors:** Ahmad Kalateh Sadati, Seyed Ziaaddin Tabei, Hamzeh Salehzadeh, Farnaz Rahnavard, Bahia Namavar Jahromi, Soroor Hemmati

**Affiliations:** 1 Department of Sociology and Social Planning, Management and Social Sciences, Shiraz, Iran;; 2 Department of Medical Ethics and Philosophy of Health, School of Medicine, Shiraz University of Medical Sciences, Shiraz, Iran;; 3 Department of Epidemiology, School of Health, Shiraz University of Medical Sciences, Shiraz, Iran;; 4 Department of Nursing, School of Nursing and Midwifary, Rasht University of Medical Sciences, Rasht, Iran;; 5 Department of Obstetrics and Gynecology, School of Medicine, Shiraz University of Medical Sciences, Shiraz, Iran;; 6 Department of Educational Management, Islamic Azad University, Marvdasht, Iran

**Keywords:** Induced Abortion, Unintended Pregnancy, Neutralization, Religion, Sociological Theory

## Abstract

**Background: **Induced abortion is not only a serious threat for women’s health, but also a controversial topic for its ethical and moral problems. We aimed to evaluate the relationship between neutralization techniques and attempting to commit abortion in married women with unintended pregnancy.

**Methods: **After in-depth interviews with some women who had attempted abortion, neutralization themes were gathered. Next, to analyze the data quantitatively, a questionnaire was created including demographic and psychosocial variables specifically related to neutralization. The participants were divided into two groups (abortion and control) of unintended pregnancy and were then compared.

**Results:** Analysis of psychosocial variables revealed a significant difference in the two groups at neutralization, showing that neutralization in the control group (56.97±10.24) was higher than that in the abortion group (44.19±12.44). To evaluate the findings more accurately, we examined the causal factors behind the behaviors of the abortion group. Binary logistic regression showed that among psychosocial factors, neutralization significantly affected abortion (95% CI=1.07-1.35).

**Conclusion:** Despite the network of many factors affecting induced abortion, neutralization plays an important role in reinforcing the tendency to attempt abortion. Furthermore, the decline of religious beliefs, as a result of the secular context of the modern world, seems to have an important role in neutralizing induced abortion.

## Introduction

Induced abortion was recognized as a problematic issue in the past decades, and according to Calderone, induced abortion has been introduced as a critically social issue.^1^ Brennan considered induced abortion a new issue in the new world.^2^ Views of scholars on this social problem are controversial, as some try to defend this practice,^3^ while others question its unethicality.^4^^,^^5^ Kaczor believes that one way to defend abortion is to defend infanticide, the intentional killing of a baby after birth.^5^ Beckwith, however, as one of the opponents of induced abortion, contends that: From the moment of conception, a fetus is a complete member of the society. Therefore, it is morally incorrect to kill it because abortion, it successful, kills a member of the society and id thus morally wrong.^4^

On the other hand, the general context of Abrahamic religions is in sharp conflict with the practice of induced abortion. With very little difference, Islam,^6^^,^^7^ Judaism,^8^^,^^9^ and Christianity^10^^-^^12^ all reject induced abortion. For instance, in Judaism “rather, normative *halakhic* positions have always held that some amount of abortion is required–in order to save the life of the mother–but have uniformly rejected abortions that cannot be justified either because of maternal need, or for a threat to the fetus, or perhaps to save another child”.^8^

Despite these oppositions against induced abortion, many social movements insist on legalizing this act. Presumably, the legalization of induced abortion is largely because of the increasing secular ideology governing the world. Secularity seems to represent the main character of the modern wisdom.

Because of its problematic nature, induced abortion has been a controversial debate in scientific domains such as philosophy, sociology, ethics, medical ethics, philosophy of ethics, health justice, while legislators follow the debates over induced abortion seriously. In these disputes, certain main topics are normally addressed including fetal rights, the right to life, legal right of the fetus, parents’ rights to determine the life of fetus, intervention in fetal rights, ethics of legal abortion, and so forth. This nature and breadth of these debates show the importance of fetal rights on the one hand, and many issues about induced abortion on the other. 

Also, induced abortion is considered an illegal act according to Iranian Civil and Jurisprudence Laws. According to the Islamic tradition, there are two exceptional cases in which abortion is considered legal: when the health of the mother is jeopardized, and when the fetus has severe chromosomal abnormalities. However, abortion in these two cases is subjected to certain limitations and cannot be simply performed. For instance, even if the validity of the cases is established, obtaining certification for abortion involves complicated medical procedures.

Despite this formal law, the percentage of induced abortion is high in Iran. Formal statistics announced an approximate number of eighty thousand attempts of induced abortion in Iran.^13^ However, the real number of attempts is much higher than that figure. For example, annually about 11,500 abortions are committed by married women in Tehran. In the year before the survey was conducted, the estimated total abortion rate was 0.16 abortions per woman, and the annual average abortion rate was 5.5 abortions per 1,000 women.^14^

In Iran, because induced abortion is a criminal act, most cases of abortion are committed illegally. There are many methods for performing abortion, while the frequently used ones include using prostaglandin injections, pills or suppositories, hitting, and using herbal medications. Still, the use of prostaglandin derivatives is prevalent especially in cases of injection. Because many induced abortions are performed under unstandardized and underground conditions, and because of the side-effects of such abortions (i.e. severe abdominal pain and vaginal bleeding), many women in these cases are referred to hospitals. Generally, many of them are discharged after hydration and cardiovascular stability examinations, except those who have a fetal tissue remaining in their uterus. These cases are taken to the operation room and undergo Dilatation and Curettage (D&C) to take the tissue out. In some underground cases, after injection, D&C procedures are performed with unsterile instruments and according to traditional methods by uneducated individuals. Sometimes these procedures can lead to uterine or bowl rupture that can lead to many serious problems that may cause irrecoverable injuries to the body. Some researchers are concerned with other problems such as infection^15^^,^^16^ and female mortality.^17^

This research views induced abortion from a critical perspective. The importance of the study is that it introduces induced abortion not only as a serious threat for women’s health, but also as a controversial topic for its ethical and moral problems. This study is an endeavor to evaluate the main factors affecting induced abortion, based on the psychosocial theory known as *Neutralization Techniques*.

Neutralization Techniques (Theoretical Framework)

Neutralization techniques, is a theory proposed in the context of the sociology of deviance. This theory was first introduced by two sociologists Matza and Syeks and their argument was: whether an act is considered as delinquency is largely dependent on the perception of the delinquent. Such that acts that are considered as delinquency from the society’s perspective might not be so in the eye of the delinquent.^18^ So, the main concern of this theory is to study the effects of neutralization techniques in juvenile delinquency. Juvenile delinquents, after committing deviant behaviors, justify their acts for themselves or others, using techniques of neutralization. In these techniques, the offender uses five methods for justification: (1) *Denial of responsibility* in which they will propose that the act is on the forces beyond our control, or mostly so, made them do it; (2) *Denial of injury* that the offender will propose that their actions did not cause any harm or damage; (3) *Denial of the victim* that they believes there were no victims; (4) Condemnation of the condemners for example they say “The police broke the laws”; and (5) Appeal to higher loyalties that the offender suggests there’s a hierarchy of moral values, such that some are more important than others, such as the protection of a friend.^18^^-^^20^ So neutralization techniques are methods that a person uses for crossing social and cultural barriers. With the use of neutralization techniques the delinquent justifies deviant motivational patterns and allows for the delinquent to engage in delinquency without any damage to his/her self-image.^21^

According to this theory, one of the important causes of deviance and delinquency is neutralization techniques. These techniques are personal justifications for any deviance including abortion. Viewed from the perspective of neutralization, induced abortion is a criminal and unethical act. In this study, we drew on the framework of the theory, with the assumption that the effective variable reinforcing attempted abortion is neutralization techniques. As a result, the main question addressed in this study is to discover any positive relationships between neutralization and abortion attempts. 

## Materials and Methods

This research was based on a sectional-analytical method that compared two groups of women with relatively equal socioeconomic status. These participants were married and all of them had legitimate and legal pregnancies. Yet, both groups had unintended pregnancy, and participants of one of the groups (abortion group) had attempted abortion, whereas participants of the second group, despite unintended pregnancy, had not attempted abortion (control group). 

The participants were selected from two government hospitals as OB/GYN centers in Shiraz, southern Iran. The first hospital was Shahid Faghihi in which the OB/GYN emergency receives many emergency cases including women who experience abortion with unstandardized methods and need follow-up care (due to severe abdominal pain and vaginal bleeding). This center accepts women who had legitimate and legal pregnancy and induced abortion. The other hospital was Hafiz, which is known as an OB/GYN center, and the midwifery clinic in the hospital provides women with pregnancy and post-pregnancy care. In this center many of pregnant women have a personal file recording their course of pregnancy and its care. This center accepts women who had unintended pregnancy but did not attempt abortion. This study lasted about 9 month from July 2012 to April 2013.Because unintended pregnancy and attempted abortion had not been reliably studied from the statistical perspective in Shiraz, and because no other relevant study concentrated on neutralization had been conducted, no specific formula was considered for selecting the sample. As a result, analysis only relied on comparing the two groups. To select the participants, quota sampling method was used. Moreover, the study was conducted by the Ethics Committee of Shiraz University of Medical Sciences and informed consent was obtained from the participants upon interviews and survey. 

In this study, before any statistical or quantitative study was carried out, in-depth interviews were conducted with some women who had experienced abortion. This study was conducted by special midwifery staff. Qualitative analysis of the interviews showed that many of the women who had experienced abortion used neutralization techniques to justify induced abortion. Thus, we started the quantitative study according to postulates of the neutralization theory. Considering the neutralization theory, the qualitative study, and consultation with experts, the questionnaire was formulated to evaluate neutralization techniques in abortion. 

The questionnaire included 14 questions that addressed 5 viewpoints about abortion based on Matza and Syeks’s neutralization theory as mentioned above. Three other psychosocial variables were also taken into account: religious orientation, self-esteem and life satisfaction. These dimensions were also put into a researcher-made questionnaire, and the face validity of the questionnaire was confirmed by experts. Each of these variables consisted of a number of indices which were 8, 10, and 5 for religious orientation, self- esteem, and life satisfaction, respectively. Also, abortion consisted of 15 indices as mentioned. The questionnaire was created based on a 5-point Likert Scale (ranging from 0 “I strongly disagree” to 5 “I strongly agree”). Reliability was also confirmed according to Cronbach’s alpha test. Results showed that the reliability of neutralization techniques, religious orientation, self-esteem, and in life satisfaction were 0.860, 0.839, 0.718, and 0.860, respectively.

Also, we tried to examine the effect of some demographic and socioeconomic factors such as level of education, family income, number of children, husband’s job, and the sex of the previous child on neutralization and neutralization techniques. After the questionnaires were compiled, the data were analyzed using SPSS software, version 19. T and chi-square tests  and one-way ANOVA were used as appropriated. 

## Results

76 women participated in the abortion group and 75 women in the control group. Because these two groups had to be compared for each of the factors under study, to perform a better evaluation, we compared the demographic factors in the two groups. According to statistical logic, these two groups had to have minimum differences as far as demographic factors were concerned. Chi-square test ‎(P<0.05) and *t* test (P<0.05) showed that both groups were similar in terms of all demographic and socioeconomic variables, except for age. So the selection of the two groups according to statistical logic is acceptable.

Also, the statistical distribution of the two groups showed a normal distribution. That is, both abortion and control groups represented relatively comparable conditions, and the distribution was statistically normal. The abortion group included some variables important for this study. For example, history of abortion, method of abortion, those who introduced abortion to the women, cause of attempted abortion from the women’s point of view, and the age of the aborted fetus (in week). Table 1, shows the frequency distribution of these variables in the abortion group. As shown, (a) many of the participants revealed that it was their first experience of abortion (76.3%); (b) use of prostaglandin derivatives is a common method in these cases (58.51%); (c) many of participants viewed the main cause of abortion to be economical problems and psychological stress (52.8%). One important point about the method of abortion is that some women had experienced multiple methods such as the use of derivatives of prostaglandin and vaginal manipulation, the use of herbals, and hitting. As the purpose of this research, 4 psychosocial variables were compared between the groups under study. In this comparison, the observed differences were evaluated. Table 2 illustrates the concerns of this research. Neutralization techniques, religious orientation, self-esteem and life satisfaction between the two groups was significantly different. To evaluate the findings more accurately, we examined the causal factors behind the behaviors of the abortion group. Binary logistic regression showed that, from among psychosocial factors, neutralization significantly affected abortion (95% CI=1.07-1.35).

**Table 1:** Frequencies of some main variable about aborted group

**Variable**	**Frequency**	**Percentage**
Previous history of abortion		
Yes	18	23.7%
No	58	76.3%
Method of abortion		
Derivatives of prostaglandin	55	58.51%
Vaginal manipulation	8	11.4%
Herbal drugs	5	7.1%
Hitting	26	29.33%
Who introduced abortion to you		
Friends	33	47.1%
Medical group	20	28.6%
Family members	14	20%
Media	3	4.3%
Why did you attempt abortion?		
Economical problems and psychological stress	38	52.8%
Shame in face of family	4	5.6%
Previous child is a girl	10	13.9%
Pressurized by husband	5	6.9%
Fear from fetus abnormalities	8	11.1%
Pregnancy without sufficient time after previous child	7	9.7%
Age of aborted fetus (weeks)	7.58±3.91	-

**Table 2:** Comparison of the 4 psychosocial variables in the two groups

**Variables**	**Abortion group** (** x̄**±**SD**)	**Control group** **(**x**=**x̄**±**SD**)**	**Significance** ** (*t* test) **
Religious orientation	30.29±5/80	32.03±4.35	0.001^**^
Self-esteem	31.82±7/59	32.21±6.43	0.000^**^
Life satisfaction	22.26±7/21	23.18±5.95	0.004^**^
Neutralization	44.19±12/44	56.97±10.24	0.000^**^

*t* test at P<0.05

To obtain a better evaluation of neutralization, we examined the effect of demographic variables on neutralization in the abortion group (table 3). As shown, husbands’ job and history of abortion significantly affected neutralization. So it can be predicted that a woman who attempts abortion is more likely to accept abortion for her future pregnancies, because of her increased neutralization. Also, one-way ANOVA analysis revealed that family income (low, average, high) and education level (illiterate, secondary education, higher education) did not have any significant relationship with abortion in the abortion group.

**Table 3:** Relationship between demographic factors and neutralization in abortion group

**Variables**	**Neutralization**	**P value**
How old are you?		0.69
<20 years old	46.66±4.16	
>20 years old	43.73±12.79	
How many children do you have?		0.52
1	43.13±9.31	
>1	44.97±14.89	
What is your job?		0.10
Housekeeper	45.44±12.28	
Employee	39.86±12.36	
What is your husband’s job?		0.004^**^
Employee of official organizations	45.60±11.95	
Self-employed	39.86±12.36	
What is the gender of your previous child?		0.4
Boy	45.08±13.4	
Girl	42.58±11.09	
Do have previous history of abortion?		0.009^**^
Yes	48.48±9.87	
No	42.86±12.92	

Sample *t* test P<0.05

## Discussion

Many factors can encourage pregnant women to commit induced abortion, whereas neutralization techniques appear to be a major factor. These factors are psychosocial variables that justify induced abortion. Neutralization techniques transform the deviant nature of the act into a normal social practice. In this study, we evaluated the effect of neutralization techniques on the tendency to attempt abortion in case of legal but unintended pregnancies. To do this, we compared two groups of legal but unintended pregnant women: the participants of the first group had not attempted abortion, whereas the participants of the other group had. 

To provide a reliable evaluation, qualitative and quantitative research methods were used. Results of the qualitative research showed that neutralization was a major motive extracted through in-depth interviews with women who had attempted induced abortion. Quantitative data also revealed a significant difference between the two studied groups. The results provide important implications for further debate and exploration. More specifically, women who had experienced unintended pregnancy had expressed meaningful neutralization for justifying abortion. Some general neutralizing quotes were: “I did it for my husband and my other living children”; “I was under unfavorable conditions financially”; “the fetus is not a complete human”; “I was forced to do it” and so on.

According to table 2, neutralization in the control group was higher than that in the abortion group. This research was, on the contrary, founded upon the assumption that neutralization in the abortion group would to be higher than that in the control group. To explain this conflict, two reasons can be considered: on the one hand, many women who had abortion had felt guilty in their first experience. This experience of guilt might have affected their view about abortion. On the other hand, according to the results, the social variable showed a significant difference between the two groups, while psychosocial factors such as self-esteem, life satisfaction, and religious orientation are important as well. This comparison implies that attempt to commit abortion is affected by multivariable factors and that any abortions have a network of causes. As mentioned, evaluation with binary logistic regression (P<0.005) showed that neutralization, from among psychosocial factors, had a significant effect on abortion (P=0.001) in the abortion group. Nevertheless, although neutralization is a major factor in abortion, abortion is a consequence of many demographic and psychosocial factors.

Also, it can be postulated that neutralization techniques are considered before the attempt to commit abortion, because after attempting abortion many women experience guilt. This experience is, of course, forgotten very soon. According to table 3, history of abortion has a significant relationship with neutralization. On the other hand, despite the experience of guilt after the attempt, guilt can be a significant factor in declining neutralization at the first attempt.

Yet, statistical results showed that the husband’s job and history of attempt had significant effects on neutralizing abortion. Apparently, women whose husbands are self-employed do not effectively prevent pregnancy, simply justifying the neutralization of abortion. Despite many changes in social classes in Iran, families living on non-official jobs usually belong to the middle or low socioeconomic classes. This condition is along with other negative factors such as poor education, poor health, and so on. Such conditions encourage families toward neutralization. Yet, this assumption should be subjected to further examination in other relevant studies. 

According to the comparisons, beside self-esteem and life satisfaction, religious orientation plays a major role in preventing induced abortion. With decline in this orientation, the chances of attempting abortion may increase, because religious traditions discourage abortion. Generally, as noted above, Abrahamic religions reject any attempt to commit abortion, with some few exceptions. In this religious context, attempted abortion is viewed as an unforgivable “sin”. Based on the results, one can claim that enriching religious views and approaches in families and especially spouses can effectively help prevent abortion.

Erfani’s research confirms the results of the present study. Erfani’s research specifically confirmed that weakness of religiosity can be a factor affecting abortion.^14^ Chinichian et al. also observed comparable results. In their qualitative study, religious beliefs were considered the main inhibitory factor against abortion.^22^ Generally speaking, the secular context of the present world represents justifications for neutralizing abortion although it is a negative practice generally viewed as a moral defect and a religious sin.

As for the role of “neutralization techniques” in abortion and its causes, we did not find any field research. Yet, Brennan has a critical theoretical paper on abortion neutralization. He believes that “A confluence of forces such as abortion legalization, advancing abortion technology, establishment of abortion as a valid medical procedure, population pressures, scarcity of resources, and quality of life concerns are several factors rationalizing abortion with considerable authenticity”.^2^ As mentioned above, the secular context at a macro level sets favorable conditions supporting the neutralization of induced abortion. To prevent this unethical act, policymakers should focus on the prevention of unintended pregnancy in families, try to develop a religious context, and consequently enrich it in macro, middle and micro levels of societies.

The present study, of course, faced some limitations which include: (a) the number of samples in the abortion group was small because most of women attempting abortion do not refer to hospitals; (b) the background of studies on neutralization and abortion was still underdeveloped at the time the study was conducted; (c) some of the samples rejected to participate in the study, although they could have enriched the population. Considering these limitations, researchers are recommended to conduct more extensive surveys in future studies. 

## Conclusion

Despite many social movements insisting on legalizing induced abortion, this act has its moral problems and medical threats. Nonetheless, it has high prevalence in many countries especially in Iran. Besides other psychosocial factors, neutralization techniques have main effect on attempts to induce abortion. Considering the results of this study from a general perspective, it can be postulated that neutralization in today’s secular context is a highly effective factor in women. So by enriching religious beliefs and psychosocial situations in families, this immoral act can be prevented. 
